# Validation of the ^2^H-SNIF NMR and IRMS Methods for Vinegar and Vinegar Analysis: An International Collaborative Study

**DOI:** 10.3390/molecules25122932

**Published:** 2020-06-25

**Authors:** Federica Camin, Marco Simoni, Armin Hermann, Freddy Thomas, Matteo Perini

**Affiliations:** 1 Center Agriculture Food Environment (C3A), University of Trento, via Mach 1, 38010 San Michele all’Adige (TN), Italy; federica.camin@unitn.it; 2 Fondazione E. Mach (FEM), Via Mach 1, 38010 San Michele all’Adige (TN), Italy; marco.simoni@fmach.it; 3 Institut für Lebensmittelchemie, Nikolaus-von-Weis-Straße 1, 67346 Speyer, Germany; Armin.Hermann@lua.rlp.de; 4 Eurofins Analytics France, 9, Rue Pierre Adolphe Bobierre, B.P. 42301, CEDEX 3, 44323 Nantes, France; FreddyThomas@Eurofins.com

**Keywords:** vinegar, balsamic vinegar, SNIF-NMR, 1H-NMR, IRMS

## Abstract

This paper presents the results of a collaborative study involving seven laboratories and concerning two samples of wine vinegar, one of apple vinegar and four of balsamic vinegar. The aim of the study was to define standard deviations of repeatability (sr) and reproducibility (sR) for vinegar and balsamic vinegar stable isotope ratios of H (D/H), C (δ^13^C) and O (δ^18^O), in order to establish them as fully recognized official standards. Acetic acid was extracted and subjected to (D/H)_CH3_ and δ^13^C analysis. δ^18^O analysis was performed on whole samples. The grape must solution remained after distillation of balsamic vinegar was fermented and the resulting ethanol was subjected to (D/H)_I_, (D/H)_II_, R and δ^13^C analysis. The sr and sR were 0.6 ppm and 1.1 ppm for (D/H)_CH3_, 0.14‰ and 0.25‰ for δ^13^C of acetic acid, 0.1‰ and 0.17‰ for δ^18^O of water, 0.19 ppm and 0.64 ppm for ethanol (D/H)_I_, 1.14 and 1.31 ppm for (D/H)_II_, 0.09 and 0.11‰ for δ^13^C of ethanol. These data are in line with those in the literature or reported in corresponding official methods, and sr and sR of balsamic vinegar are in line with those of vinegar and must.

## 1. Introduction

According to European Regulation 479/2008 [1], vinegar is defined as a product obtained exclusively from the acetous fermentation of the declared sources (grape for wine and balsamic vinegar, including the IGP ‘Aceto Balsamico di Modena’ and apple for apple vinegar). Thus acetic acid cannot be obtained from either petroleum derivatives or wood pyrolysis (synthetic acetic acid) or from the fermentation of exogenous sugars (e.g., from beet or cane). In addition, vinegar cannot be produced from dried grapes or fruit juices diluted with water (e.g., the ‘raisin vinegar’, commonly produced in some Mediterranean countries).

Isotopic methods have been recognized by the European Committee for Standardization (CEN) and in part by the Organisation Internationale de la vigne et du vin (OIV) as a means of detecting the non-permitted presence of exogenous acetic acid and water in vinegar (CEN) and specifically wine vinegar (OIV). The methods used are EN 16466-1 for D/H in the methyl site of acetic acid [(D/H)_CH3_] using ^2^H-SNIF-NMR (Site Specific Natural Isotope Fractionation-Nuclear Magnetic Resonance), EN 16466-2 and OIV 510/2013 for analysis of ^13^C/^12^C in acetic acid (δ^13^C ‰) using IRMS (Isotope Ratio Mass Spectrometry), and EN 16466-3 and OIV 511/2013 for analysis of ^18^O/^16^O in water (δ^18^O ‰) using IRMS.

The OIV method for acetic acid (D/H)_CH3_ using ^2^H-SNIF-NMR (CII SCMA 2013-03 13 FV 1415) is at stage 5 (OIV method OENO-SCMA 13-527) but is not yet validated based on a collaborative study.

Moreover, recently it was experimentally proven that OIV and CEN methods are also applicable to the analysis of acetic acid extracted from balsamic vinegar (D/H, δ^13^C) as well to the ethanol fractions obtained by distillation after fermentation of cooked must of balsamic vinegar (D/H, δ^13^C) and to the water fraction (δ^18^O) [2]. Furthermore, in this case, the OIV and CEN vinegar and must methods (methods OIV-MA-AS311-05 for D/H, OIV-MA-AS312-06 for δ^13^C and OIV-MA-AS2-12 for δ^18^O) need validation for implementation with balsamic vinegar ingredients.

This collaborative study involved different laboratories and concerned two wine vinegar samples, one apple vinegar sample and four balsamic vinegar samples (two from wine vinegar, one from apple vinegar and one from cane distillate vinegar), in double blind duplicates, for a total number of 14 samples. The test was performed according to the International Union of Pure and Applied Chemistry (IUPAC) protocol [3] and International Organisation for Standardisation (ISO, Geneva, Switzerland) Standards 5725/2004 and 13528/2005. Acetic acid was extracted from samples of both vinegar and balsamic vinegar, purified by distillation and subjected to (D/H)_CH3_ analysis using SNIF-NMR and to δ^13^C analysis using IRMS. Vinegar and balsamic vinegar were also subjected to δ^18^O analysis using IRMS. Additionally, the aqueous solution containing the must (sugar fraction) from balsamic vinegar was fermented and the ethanol obtained was extracted and subjected to (D/H)_I_, (D/H)_II_, R and δ^13^C analyses.

Nine laboratories were contacted for the collaborative study, but only seven (see below) sent the results (Table 1 and Table 2). Only four laboratories performed all the analyses on all the ingredients (acetic acid, ethanol, vinegar and balsamic vinegar water).

The aim of the study was to define the validation parameters for (D/H)_CH3_ of acetic acid from vinegar and for the stable isotope ratios of acetic acid (D/H and δ^13^O), ethanol (D/H, δ^13^O) and water (δ^18^O) of balsamic vinegar.

## 2. Results and Discussion

### 2.1. Pre-Trial Study

Before starting the collaborative study, a pre-trial study was performed in order to detect the correct intervals of integration for both TMU (tetramethylurea) and acetic acid in 1H-NMR acquisition useful for manual integration.

Each participant received a tube with a solution of acetic acid and water, ready to be analyzed. Purity of acetic acid (84.9% *w/w* acetic acid), weight of TMU(g) and weight of acetic acid solution (g) were given to each participant (see Table 1).

First of all (Step 1), the participants had to identify, using their instrumental conditions, the best integral widths for TMU and acetic acid (express in Hz) to guarantee the correct TMU/acetic acid molar ratio to achieve the reported purity value of acetic acid (84.9%).

Afterwards (Step 2), participants had to report, if known, the integral widths of TMU and acetic acid (in Hz) normally used by the lab and/or fixed by the software and the TMU/acetic acid molar ratio obtained by the intervals of integration

The (D/H)_CH3_ computed on the basis of the weight of pure acetic acid [(D/H)_CH3_ mass] and in Step 2, also using the combined ^1^H and SNIF-NMR experiment [(D/H)_CH3_ proton] was provided by the participants (Table 1).

By comparing the results of the two steps, the intervals of integration are the same in the two steps for some laboratories and, when different, are larger in Step 2. The NMR molar ratio was the same between laboratories in Step 1, whereas in Step 2 some differences are evident. In particular, for laboratories E and F, purity (% of acetic acid) is lower as they found some residue of the ether used for the extraction of acetic acid inside the solution. In spite of this, we did not observe significant differences (*p* < 0.01) between the (D/H)_CH3_ values of the two steps (different intervals of integration), nor between the values computed on the basis of the weight of pure acetic acid [(D/H)_CH3_ mass] and those computed using the combined ^1^H and SNIF-NMR experiment [(D/H)_CH3_ proton], or for the different purities found in the solutions. Laboratory 7 is an exception, as in both steps the standard deviation is very high (between 2.3 and 2.4) and in Step 2 the D/H value is higher. It was therefore considered an outlier (Table 1).

Excluding this lab, (D/H)_CH3_ values ranged between 121.3 ppm and 122.8 ppm and the standard deviation between 0.2 and 0.9, which is in line with the literature [4].

On this basis, it was decided not to fix and establish an integration interval for the collaborative study. Each laboratory was required to perform the analyses using its own routine integration intervals.

### 2.2. Collaborative Study

Seven laboratories presented their results for (D/H)_CH3_ of acetic acid, five laboratories for δ^13^C of acetic acid, four for δ^18^O of vinegar and balsamic vinegar, five for (D/H)_I_ and four for δ^13^C of ethanol of balsamic vinegar must, after fermentation. None of the laboratories was eliminated as a technical outlier, although some of them did not implement the exact protocol.

The data received from the participants are summarized in Table 2.

As required in the IUPAC harmonized protocol for collaborative studies on complex methods [3], the minimum of five laboratories supplying valid results was satisfied for all parameters except for vinegar and balsamic vinegar δ^18^O and for ethanol δ^13^C. The standard deviations of repeatability (sr) and reproducibility (sR) for each sample were calculated considering only the valid results of the blind duplicates (see Data Elaboration section) according to the ISO Standard 5725 and the IUPAC protocol [3]. A summary of the results of these calculations is presented in Table 2.

In general, the sr and sR values obtained for all materials are comparable, irrespective of starting material (wine, apple, cane sugar), of different extracting solvents, of the presence of residue of ether found by some laboratories, or of the use of different integration software.

For acetic acid (D/H)_CH3_, the average standard deviation of repeatability (sr) was 0.6 ppm and that of reproducibility (sR) was 1.1 ppm. The sr and sR values ranged from 0.33 to 0.85 ppm and from 0.61 to 1.61 ppm, respectively, and they were comparable with those of CEN method EN 16466-1.

The average standard deviations of repeatability (sr) and reproducibility (sR) were respectively 0.14‰ and 0.25‰ for acetic acid δ^13^C, 0.1‰ and 0.17‰ for vinegar and balsamic vinegar δ^18^O, 0.19 ppm and 0.64 ppm for ethanol (D/H)_I_, 1.14 and 1.31 ppm for (D/H)_II_, 0.09 and 0.11‰ for ethanol δ^13^C. All of these data are in line with those reported in the corresponding OIV and CEN methods (EN 16466-2 and OIV 510/2013, EN 16466-3 and OIV 511/2013 for acetic acid, OIV-MA-AS311-05, OIV-MA-AS312-06 and OIV-MA-AS2-12 for ethanol).

The standard deviation of repeatability and reproducibility values of balsamic vinegar are in line with those of vinegar. This means that the officially recognized methods for vinegar and must can be also applied to the ingredients of balsamic vinegar, with the same validation parameters [2].

## 3. Materials and Methods

### 3.1. Samples

For the pre-trial experiment, a tube with a solution of acetic acid and water watables was prepared for each participant. A sample of 100% pure acetic acid glacial (≥99%, ReagentPlus^®^, Merck KGaA, Darmstadt, Germany) was diluted with ultrapure distilled water (15% *w/w*) and then submitted to the whole procedures of extraction and distillation described below. Additionally, each participant was given the values of purity of acetic acid (84.9% *w/w* acetic acetic, determined on the basis of 1H-NMR), weight of TMU(g) and of acetic acid (g) (see Table 1).

Vinegar and balsamic vinegar samples were produced and provided by Ponti s.p.a. (Ghemme, Novara, Italy) and included: 2 wine vinegar samples, 1 apple vinegar sample and 4 balsamic vinegar samples (2 from wine vinegar, 1 from apple vinegar and 1 from cane sugar vinegar). The 7 samples were sent to the laboratories in blind duplicates, thus each laboratory received 14 samples. This procedure complies with the requirements of the IUPAC internal harmonized protocol for collaborative studies [3], which asks for more than five ‘materials’ (i.e., different matrix/sample pairs). 

### 3.2. Pilot Production of Vinegar and ABM 

The raw materials (wine, concentrated grape must, apple juice and cane molasses) were supplied by producers who guaranteed their authenticity. 

Wine, apple and cane sugar vinegars were produced starting directly from wine, apple juice and cane molasses, whereas balsamic vinegars were produced by adding the same cooked grape must to wine, apple and cane sugar vinegar.

Wines and apple juices were transformed into vinegars using two fully automated pilot fermenters (CIP/SIP Fermenters, SYSBIOTECH GmbH, Vienna, Austria) (each with a capacity of 8 L) and a continuous fermentation process (at least 10 days per sample) identical to the industrial process with respect to time, temperature and concentration of alcohol and acetic acid (40 h to entirely transform the alcohol into acetic acid, 34−36 °C, 10.0−10.4% of alcohol in wine, transformation efficiency 95−97%). Oxidation of ethanol was performed by means of Acetobacter spp. bacteria (Merck KGaA, Darmstadt, Germany)

The ABM (Aceto Balsamico di Modena) was produced according to EU Reg. 583/2009 using pilot vacuum equipment normally used in jam production for concentration purposes.

Tangential filtration was carried out by passing the vinegar through a polysulphone membrane with a porosity of 0.6 μm; the maximum temperature throughout the process was below 37 °C. 

The content in acetic acid, expressed as acidity % *w/w*, was calculated following the official OIV method RESOLUTION OENO 52-2000. 

### 3.3. Participants

The following reference people and laboratories participated in the collaborative study:

1. Dr. Zedda Claudia, Agenzia delle Dogane, Laboratorio Chimico di Torino, Corso Sebastopoli 3, 10134 Torino, Italy
2. Dr. Diana Costinel/Oana Botoran, Stable Isotope Laboratory, National Research and Development Institute for Cryogenic and Isotopic Technologies – ICSI Rm. Valcea, Uzinei Street No. 4, Post code 240050, Romania
3. Dr. Freddy Thomas, Eurofins Analytics France, 9, Rue Pierre Adolphe Bobierre, B.P. 42301, F-44323 NANTES Cedex 3, France
4. Dr. Rebecca Kokkinofta-Diogenou, State General Laboratory, 44 Kimonos str., 2081 Nicosia, Cyprus
5. Mrs. Miriam Schmidt, Chelab Dr. Ara, Carl-Zeiss-Strasse 16, 30966 Hemmingen, Germany
6. Dr. Armin Hermann, Institut für Lebensmittelchemie, Nikolaus-von-Weis-Straße 1, D-67346 Speyer, Germany
7. Dr Matteo Perini/Federica Camin, Fondazione Edmund Mach, via Mach 1, 38010, San Michele all’Adige (TN), Italy

Details of the 7 laboratories are summarized in Table 3.

### 3.4. Methods

The analytical protocol (described below) was sent to the participants, but some laboratories adopted slightly modified approaches (see Table 3) e.g., one lab used tert. butyl methyl ether instead of diethyl ether to extract acetic acid. The instrument model/producer and standards used by the participants are summarized in Table 3. 

### 3.5. Preparation of Samples 

#### 3.5.1. Extraction and Purification of Acetic Acid

**Figure 1:**
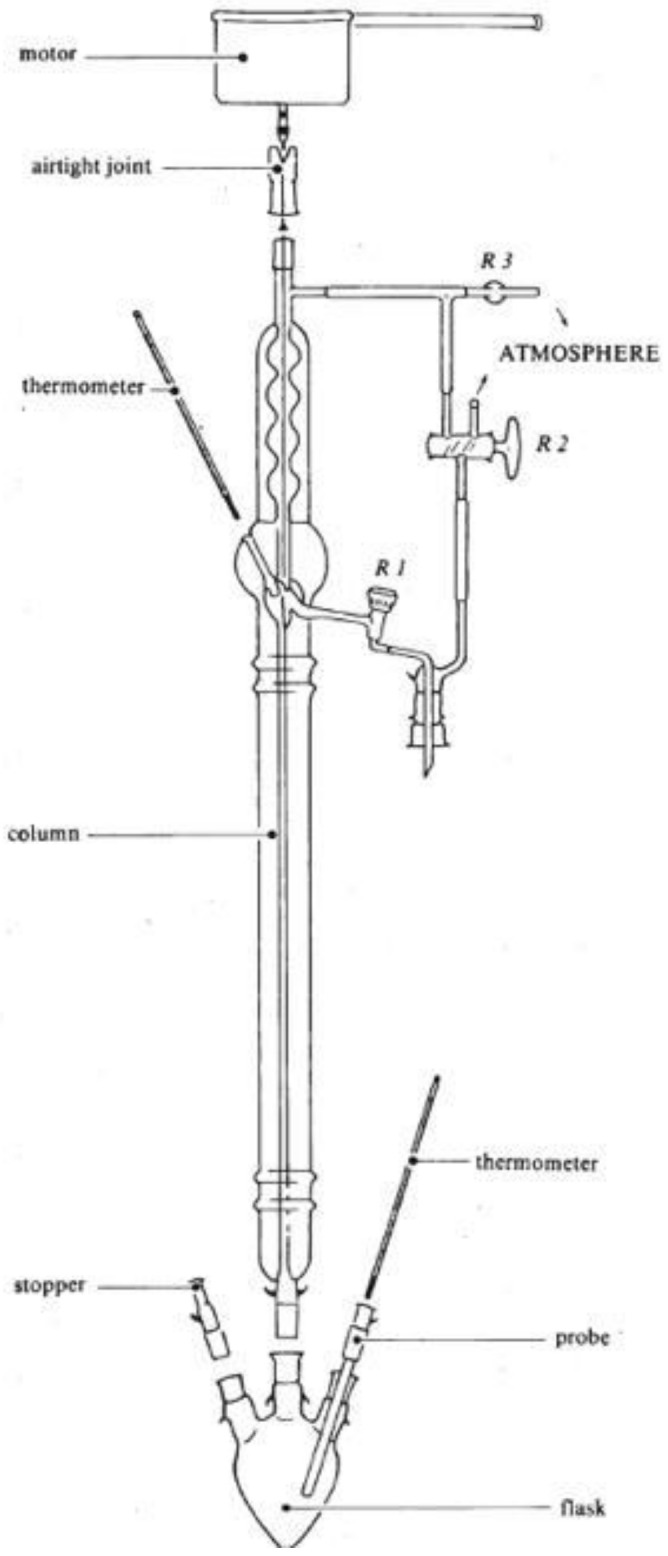
Diagram of the distillation device [from Thomas and Jamin, 2009].

Acetic acid was first extracted from vinegar and balsamic vinegar with ether and then purified by distillation. As reported in Table 1, one of the laboratories used TBME (tert. butyl methyl ether) instead of ethyl ether. In the following description the mentioned solvent is ether. At least 6 mL of pure acetic acid must be recovered at the end of the extraction. Any method that does not involve isotopic fractionation may be used to extract and purify the acetic acid. The following method (including the reagents used) and Cadiot column (Eurofins Analytics France, Nantes, France) in Figure 1 are given as an example.

#### 3.5.2. Liquid–Liquid Extraction

Around 125 mL of diethyl ether is poured into a 250 mL round-bottom flask. A 400 mL or 800 mL liquid–liquid extractor is used, depending on the vinegar acetic acid content (at least 6 mL of pure acetic acid must be recovered at the end of the extraction).

Vinegar or balsamic vinegar is poured into the column of the extractor and topped up with diethyl ether. The round-bottom flask is placed in the heating mantle connected to the extractor and water for the condenser is provided. The extraction must last at least 5 h.

After this time period, the aqueous and organic solutions contained in the extractor are separated. The organic solution is added to the extract in the round bottom flask.

In the case of balsamic vinegar, the aqueous solution containing the must has to be retrieved in a flask for fermentation, paying attention to recover only the aqueous phase. Indeed, the presence of ether with acetic acid as residue precludes fermentation.

#### 3.5.3. Purification of the Extract

The acetic acid contained in the round bottom flask in ether solution is distilled using a column, which avoids isotopic fractionation of acetic acid, as shown in Figure 1. 

An appropriate 250 mL flask is used to collect the distillate. The temperature during distillation of the ether (which has a boiling point of 34 °C) must be kept under control. When most of the ether has been distilled (there is no more vapor at the top of the column), the temperature can be increased. The distillation is complete when the internal temperature at the top of the column is stable at around 90–95 °C (pure acetic acid distils at 116–117 °C). The remaining traces of ether in the acetic acid must be removed by blowing N_2_ or air for 10 minutes on the residue at room temperature.

#### 3.5.4. Fermentation of the Aqueous Solution (for Balsamic Vinegar Only)

The aqueous solution with the sugars fraction contained in the flask for fermentation is warmed up at 35 °C for 2 h to remove any trace of ether. Then, the flask is left under a hood for one night and finally it is fermented following the official OIV-MA-AS-311-05 method. After fermentation, ethanol is recovered by distillation following the OIV-MA-AS-311-05 method.

### 3.6. Analysis

Preliminary note: the amount and type of reagents and the instrumental conditions depend on the type of apparatus used. The procedure described here is merely an example.

#### 3.6.1. Stable Isotope Analysis of Ethanol and of δ^13^C of Acetic Acid

The official OIV-MA-AS-311-05 and OIV-MA-AS312-06 methods have to be followed for (D/H)_I_, (D/H)_II_, R and for δ^13^C analysis, respectively. The EN 16466-2 or OIV 510/2013 methods have to be followed for δ^13^C analysis of acetic acid.

#### 3.6.2. Analysis of Vinegar and Balsamic Vinegar δ^18^O 

EN 16466-3 and OIV 511/2013 were followed for the analysis of ^18^O/^16^O in water (δ^18^O ‰) using IRMS.

#### 3.6.3. Analysis of Acetic Acid (D/H)_CH3_ using SNIF-NMR 

All the stages must be carried out without any significant evaporation of acetic acid, which would change the isotopic composition of the sample.

##### Preparation of the Acetic Acid Sample for NMR Measurement 

In a pre-weighed glass vial, weigh 3.25 g or mL of the solution containing acetic acid; then add 1.1 g or mL of internal standard TMU (tetramethylurea). Depending on the type of spectrometer and probe used, add a sufficient amount of hexafluorobenzene as a field-frequency stabilization substance (lock) (for example 0.15 mL for 7.05T magnet). These values are indicative and the actual volume should be adjusted according to the sensitivity of the NMR apparatus. During the probe preparation operations and until the NMR measurement is carried out, the operator must ensure that the acetic acid and TMU do not evaporate, as this would result in isotope fractionation. The sample is homogenized by shaking and then poured into the NMR tube with a diameter of 10 mm. If necessary, 0.45 μm filter fitted to a syringe is used.

##### Acquisition of the NMR Spectrum of Acetic Acid

The analysis is carried out by ^1^H-NMR and ^2^H-SNIF-NMR using a composite NMR experiment [5,6]. The ^1^H-NMR experiment is used to determine the weight ratio between tetramethylurea (TMU) and acetic acid, which is then used, together with the results from the ^2^H-SNIF-NMR experiment, to calculate the (D/H)_CH3_ isotope ratio (see an example of spectra in Supplementary Figure S1 (proton) and Figure S2 (deuterium)).

The following conditions are recommended when acquiring the ^2^H-SNIF-NMR spectrum:

1. Probe temperature must be constant at 303 K;
2. The sample should be rotated at 15 Hz;
3. Acquisition time of 6.8 s at a spectral width of about 20 ppm;
4. 90° pulse angle;
5. Fix the offset 01 at 5.1 ppm;
6. Quadratur detection mode;
7. Set O2 to 2.5 ppm.

The sensitivity (signal-to-noise ratio) for the ^2^H experiment, measured with an exponential multiplying factor LB equal to 2.0 Hz, must be greater than or equal to 150 for the methyl signal of acetic acid containing less than 25% water. If it is less, increase the number of scans.

The resolution measured on the spectrum for ^2^H experiment, transformed without exponential multiplication (i.e., LB = 0) and expressed as the width at half height of the methyl signal of acetic acid, must be less than 0.7 Hz. Use at least 48 dummy scans to ensure thermal equilibrium before the start of the experiment.

The conditions suggested for obtaining the ^1^H-NMR spectrum are as follows:

- Probe temperature and sample rotation must be the same as for 1H-NMR;
- Acquisition time of at least 4.1 s (the sum of AQ and D1 must be at least 11 s);
- Spectral width at least 16 ppm;
- Pulse angle of 30° or lower;
- D1 = 7 s (the sum of AQ and D1 must be at least 11 s);
- Quadratur detection mode;
- Set O1p to 5.1 ppm;
- The NS should be 8 at least;
- Use at least 8 dummy scans.

These two experiments are run on the same tube, with the ^2^H-NMR experiments running first, followed by the ^1^H-NMR experiments, or vice versa.

##### Calculation of Results of Acetic Acid 

Appropriate software based on a complex signal processing algorithm determined by the least squares method must be used to determine the area of the signal. In the absence of significant phase and/or baseline errors, other software (based on regular integration) may be used as well.

For each of the ^1^H-NMR spectra, calculate the R_H_ ratio as follows:

R_H_ = S _TMU_/S _acetic acid_ (1)

where S is the area of the ^1^H-NMR signal processed with a line broadening factor (LB) equal to 0.5 Hz. 

For each of the ^2^H-SNIF-NMR spectra, calculate the R_D_ ratio as follows:

R_D_ = S’ _acetic acid_/S’ _TMU_ (2)

where S’ is the area of the ^2^H-SNIF-NMR signal, provided by the data processing software according to the Fourier-transformed Free Induction Decay, with a bandwidth equal to 2 Hz.

Finally calculate the (D/H)_CH3_ (ppm) as follows:

(D/H) _CH3_ = R_H_ * R_D_ * (D/H)_TMU_ (3)

where (D/H)_TMU_ is the isotope ratio of the internal standard (TMU) indicated on the certificate issued by IRMM. 

Calculate the average (D/H)_CH3_ and standard deviation.

Optional software enables these calculations to be performed online.

For the pre-trial study, where the mass of pure acetic acid was known, (D/H) _CH3_ was detected also as follows [4]:

(D/H)_CH3_= Pst/Paa × Maa/Mst × mst/maa × Saa/Sst × (D/H)st (4)

where ‘aa’ is acetic acid; ‘st’, the internal standard TMU; ‘P’, the number of equivalent deuterium positions for the considered molecular site; ‘M’, molecular weight; ‘m’, weighted mass; ‘S’, NMR signal area and (D/H)st(ppm), certified deuterium content of TMU.

##### Quality Control of the Analyses

The sensitivity and resolution of the spectrometer must be checked in accordance with the specifications of the apparatus:

SD RH < 0.030 between the ^1^H-NMR spectra, where RH is the ratio between the area of the 2 signals.

SD (D/H)_CH3_ < 1.2 ppm between the ^2^H-NMR spectra.

## 3.7. Statistical Evaluation of the Data

Statistical calculations were then performed according to the IUPAC protocol [3]. Outliers were removed in the following way: a loop of Cochran tests for the removal of laboratories with the highest variance, single and pair value Huber tests for individual or paired individual outliers, then returning to the Cochran test etc., keeping a proportion of outliers <2/9. The standard deviations (SD) of repeatability (sr) and reproducibility (sR) were calculated as follow: we found the mean of the data obtained from the single lab; for each data point we found the square of its distance to the mean; we summed the value and divided it by the number of data points. At the end we took the square root and we calculated the mean of the SD.

## 4. Conclusions

Standard deviation of repeatability and reproducibility results are now available for ^2^H-SNIF NMR analysis of acetic acid (D/H)_CH3_ of vinegar and balsamic vinegar, for acetic acid δ^13^C of balsamic vinegar, for δ^1^C and D/H of ethanol resulting from the fermentation of balsamic vinegar, after removal of acetic acid and for balsamic vinegar δ^18^O. These values can be used to fully validate these methods in order to be recognized as OIV and CEN official standards for the authenticity tests of these products.

## Figures and Tables

**Table 1:** Results of pre-trial study.

Participant	Lab A	Lab B	Lab C	Lab D	Lab E	Lab F	Lab G
weight TMU(g)	1.0422	1.0428	1.0406	1.0428	1.0495	1.0431	1.0470
mmol TMU	8.9721	8.9773	8.9583	8.9773	9.0350	8.9799	9.0134
weight of acetic acid solution (g)	3.3806	3.3192	3.3698	3.3730	3.3707	3.3683	3.3575
**Step 1**							
Interval of integration of TMU (Hz)	1249–997	1489–1205	1200–1040	Lorentzian calculation	Lorentian calculation	1200–1041	n.g.
Interval of integration of acetic acid (Hz)	942–673	1157–904	1000–615	Lorentzian calculation	Lorentian calculation	973–570	n.g.
NMR molar ratio TMU/acetic acid	1.3334	1.3072	1.3297	1.3280	1.3187	1.3247	1.3166
mmol of acetic acid	47.854	46.940	47.648	47.687	47.658	47.582	47.468
weight of pure acetic acid (g)	2.874	2.819	2.861	2.864	2.862	2.857	2.850
% w acetic acid	85.00	84.92	84.91	84.90	84.90	84.83	84.90
(D/H)_CH3_ mass using % w acetic acid	122.80	121.32	122.16	121.80	122.74	122.53	121.40
SD Mass	0.20	0.91	0.60	0.90	0.69	2.34	0.40
**Step 2**							
Interval of integration of TMU (Hz)	1249–997	1699–1206	1245–990	Lorentzian calculation	Lorentian calculation	1300–1000	n.g.
Interval of integration of acetic acid (Hz)	942–673	1215–687	968–648	Lorentzian calculation	Lorentian calculation	927–636	n.g.
^1^H NMR molar ratio TMU/acetic acid	1.3334	1.3078	1.3167	1.3242	1.3207	1.3095	1.2984
mmol of AcOH	47.854	46.961	47.182	47.549	47.728	47.036	46.812
Weight of pure acetic acid (g)	2.874	2.820	2.833	2.855	2.866	2.825	2.811
% w acetic acid	85.00	84.96	84.08	84.65	85.03	83.86	83.73
(D/H)_CH3_ Proton	122.57	121.29	122.10	122.44	123.09	124.06	122.70
SD Proton	0.20	0.91	0.70	0.95	0.69	2.37	0.40
(D/H)_CH3_ Mass	122.80	121.32	122.63	122.42	122.55	124.05	121.40
SD Mass	0.20	0.91	0.61	0.90	0.69	2.37	0.40

n.g: not given.

**Table 2:** Results of the study.

**(D/H)_CH3_ ppm ACETIC ACID**							
**Sample Description**	**Wine 1**	**Wine 2**	**Apple Cider**	**ABM wine 1**	**ABM wine 2**	**ABM Apple**	**ABM Cane**
**number of valid results**	7	7	7	6	6	7	7
**number of replicates**	2	2	2	2	2	2	2
**Mean**	104.94	103.46	100.84	104.73	103.33	100.51	109.1
**sr**	0.76	0.57	0.33	0.47	0.31	0.85	0.71
**sR**	1.13	1.07	1.49	0.63	0.61	1.61	0.91
**δ^13^C ‰ vs V-PDB ACETIC ACID**							
**sample description**	**wine 1**	**wine 2**	**apple cider**	**ABM wine 1**	**ABM wine 2**	**ABM apple**	**ABM cane**
**number of valid results**	4	5	5	4	5	5	4
**number of replicates**	2	2	2	2	2	2	2
**Mean**	−26.46	−27.55	−27.64	−26.15	−27.38	−27.22	−14.30
**sr**	0.16	0.14	0.09	0.12	0.08	0.18	0.21
**sR**	0.19	0.31	0.24	0.16	0.24	0.40	0.21
**δ^18^O ‰ vs V-SMOW ACETIC ACID**							
**sample description**	**wine 1**	**wine 2**	**apple cider**	**ABM wine 1**	**ABM wine 2**	**ABM apple**	**ABM cane**
**number of valid results**	4	4	3	3	3	4	4
**number of replicates**	2	2	2	2	2	2	2
**Mean**	1.21	1.11	−4.92	1.33	1.37	−2.13	−4.48
**sr**	0.09	0.08	0.04	0.15	0.11	0.13	0.13
**sR**	0.29	0.21	0.08	0.13	0.10	0.19	0.18
**(D/H)_I_ ppm ETHANOL**							
**sample description**	**ABM wine 1**		**ABM wine 2**		**ABM apple**		**ABM cane**
**number of valid results**	5		4		6		5
**number of replicates**	2		2		2		2
**Mean**	104.47		104.90		104.57		105.03
**sr**	0.19		0.05		0.21		0.30
**sR**	0.88		0.47		0.78		0.43
**(D/H)_II_ ppm ETHANOL**							
**sample description**	**ABM wine 1**		**ABM wine 2**		**ABM apple**		**ABM cane**
**number of valid results**	4		4		5		5
**number of replicates**	2		2		2		2
**Mean**	127.95		128.58		128.92		128.68
**sr**	1.08		0.93		1.28		1.28
**sR**	1.04		0.99		1.35		1.84
**δ^13^C ‰ vs V-PDB ETHANOL**							
**sample description**	**ABM wine 1**		**ABM wine 2**		**ABM apple**		**ABM cane**
**number of valid results**	4		4		4		4
**number of replicates**	2		2		2		2
**Mean**	−26.39		−26.36		−26.35		−26.33
**sr**	0.08		0.08		0.05		0.13
**sR**	0.06		0.15		0.08		0.15

**Table 3:** Participants, instrument (model/producer) and standards used and deviations to the protocol.

Lab.	Instrument Model	Year Model	Producer	Standards Used	Deviation to the Protocol
Chelab Dr. V. Ara GmbH & Co. KG, GE	FT-NMR AVANCE III 400	2012	BRUKER (a)	TMU STA-003m (b)	For purification of the acetic acid extract, Liebig condenser was used instead of vertical distillation column.
Landesuntersuchungsamt, Institut für Lebensmittelchemie und Arzneimittelprüfung, GE	FT-NMR AVANCE 400	2003	BRUKER (a)	TMU STA-003m (b)	Use of TBME (tert. butyl methyl ether) instead of ether to extract ethanol
AGENZIA DELLE DOGANE, IT	AVANCE III 400	2008	BRUKER (a)	working standard TMU calibrated against STA-003m (b) (D/H) value 128,39	NO
ICSI Analytics Group, RO	SNIF-NMR Ascend 400/Avance III	2011	BRUKER (a)	TMU STA-003m (b)	NO
Eurofins Analytics, FR	FT-NMR AVANCE III 400	2014	BRUKER (a)	TMU STA-003m (b)	Yes, Eurospec: automatic identification
State General Laboratory, CY	SNIF-NMR AVANCE III HD 400	Magnet 2002 and console 2017	BRUKER (a)	TMU STA-003m (b)	NO
FEM, IT	FT-NMR AVANCE III 400	2008	BRUKER (a)	TMU STA-003m (b)	NO

^a^ Billerica, Massachusetts, USA; ^b^ European Commission, Joint Research Centre, Institute for Reference Materials and Measurements, B-2440 Geel, Belgium.

## Supplementary Materials

The following are available online. Figure S1. Example of a proton SNIF-NMR spectra. Figure S2. Example of a deuterium SNIF-NMR spectra.
