# Supplementary figures and images for: Validation of the 2H-SNIF NMR and IRMS Methods for Vinegar and Vinegar Analysis: An International Collaborative Study
Source: Molecules. 2020 Jun 25;25(12):2932. doi: 10.3390/molecules25122932 (PMC7356488; doi:10.3390/molecules25122932)

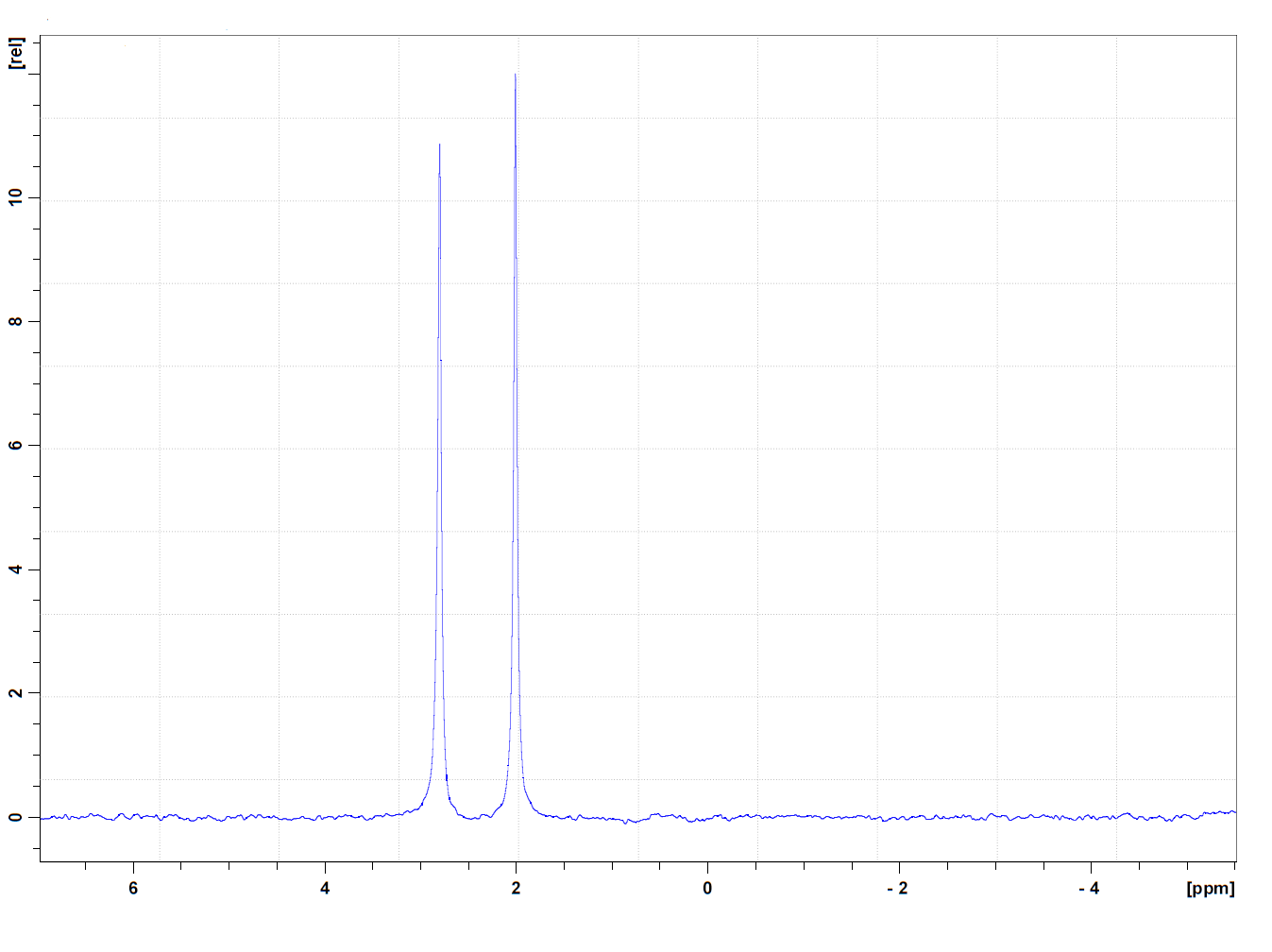

Supplement: Supplementary file 1 [file molecules-25-02932-s001.zip › Figure S2.png]

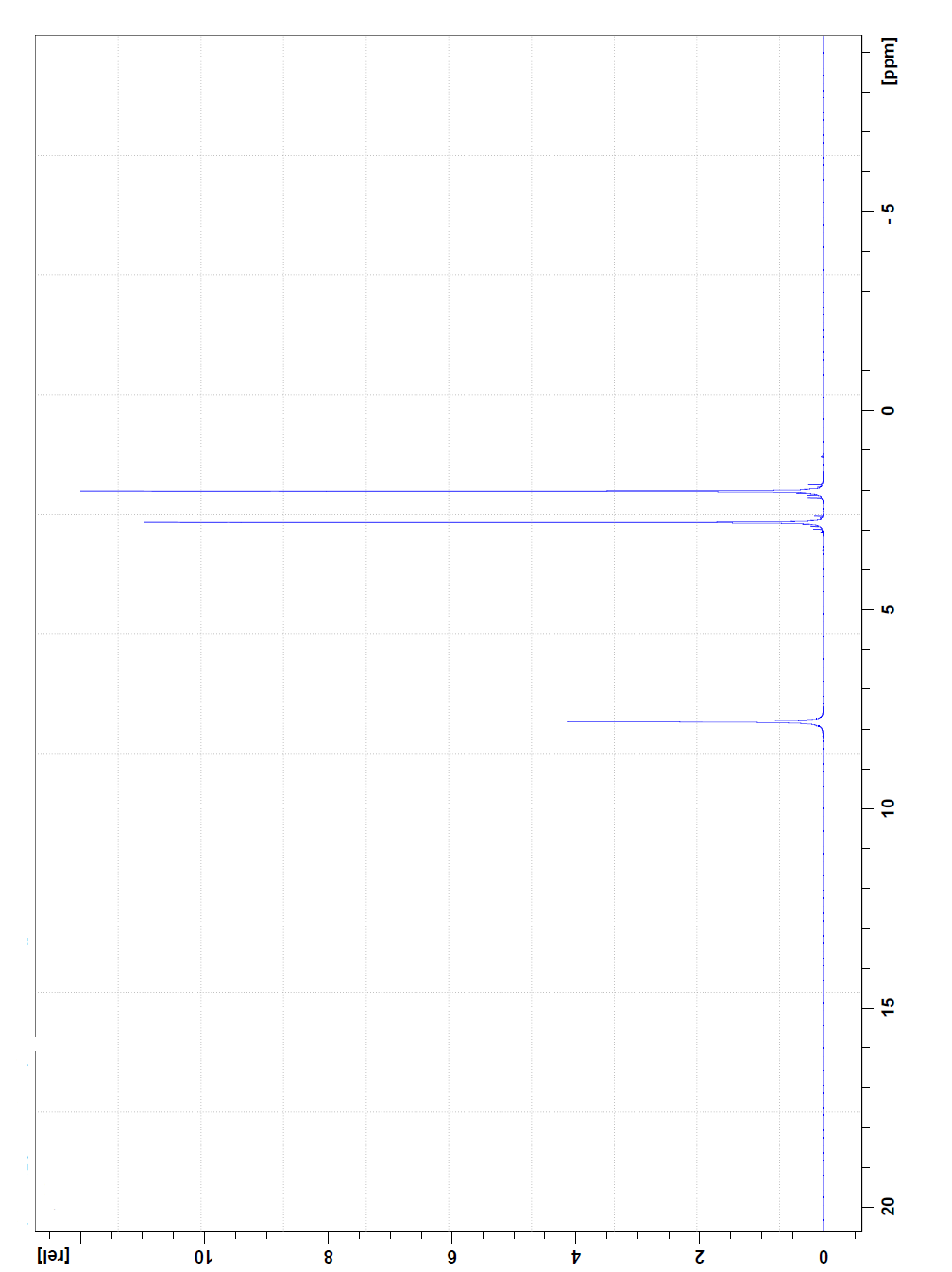

Supplement: Supplementary file 1 [file molecules-25-02932-s001.zip › Figure S1.png]
